# Coronary Artery Calcification: From Molecular Mechanisms to Interventional Strategies

**DOI:** 10.3390/ijms27115113

**Published:** 2026-06-05

**Authors:** Stefan Zivkovic, Vojko Misevic, Kosta Krupnikovic, Aleksa Obradovic, Stefan Timcic, Aleksandar Mandic, Stefan Juricic, Jelena Rakocevic, Milovan Bojic, Milan Dobric

**Affiliations:** 1 Institute for Cardiovascular Diseases “Dedinje”, 11000 Belgrade, Serbia; misevic.vojko@gmail.com (V.M.); krupnikovick@gmail.com (K.K.); obradovicaleksa96@gmail.com (A.O.); stefan.timcic@icloud.com (S.T.); aleksandar.mandic1993@gmail.com (A.M.); milovan.bojic@institutdedinje.org (M.B.); 2 Cardiology Clinic, University Clinical Center of Serbia, 11000 Belgrade, Serbia; stefan.juricic@gmail.com; 3 Institute of Histology and Embryology “Aleksandar Dj. Kostic”, Faculty of Medicine, University of Belgrade, 11000 Belgrade, Serbia; jelena.rakocevic@med.bg.ac.rs; 4 Faculty of Medicine, University of Belgrade, 11000 Belgrade, Serbia

**Keywords:** coronary artery calcification, plaque morphology, intravascular imaging, calcium-modifying strategies

## Abstract

Coronary artery calcification (CAC) represents a clear sign of advanced atherosclerosis and a strong indicator of coronary artery disease burden and cardiovascular risk. Beyond its established prognostic value, CAC significantly influences plaque biology, lesion morphology, and the technical complexity of percutaneous coronary intervention (PCI). This review summarizes current knowledge on the mechanisms of vascular calcification, its clinical determinants, diagnostic assessment, and therapeutic implications. Vascular calcification is now understood as an active, regulated process involving osteogenic transdifferentiation of vascular smooth muscle cells, inflammatory signaling pathways, extracellular vesicle release, and disturbances in mineral metabolism. Distinct calcification phenotypes exert different effects on plaque stability: micro- and spotty calcifications are frequently linked to plaque vulnerability, whereas dense, sheet-like calcification is more typical of stable fibrocalcific lesions. The prevalence of CAC increases with age and differs between sexes, while cardiometabolic risk factors, chronic kidney disease, systemic inflammation, and genetic predisposition further contribute to its development. Noninvasive computed tomography remains the cornerstone for CAC detection and quantification, enabling reliable cardiovascular risk stratification. Intravascular imaging techniques, particularly intravascular ultrasound and optical coherence tomography, provide detailed characterization of calcified plaque morphology and support optimal procedural planning. In patients with heavily calcified lesions, intravascular imaging-guided lesion preparation and stent optimization represent the most effective strategy for improving PCI outcomes.

## 1. Introduction

Coronary artery disease (CAD) is a pathological process characterized primarily by atherosclerosis of the epicardial coronary arteries, leading to impaired myocardial perfusion and a spectrum of clinical manifestations ranging from stable angina to acute coronary syndromes and heart failure [1]. The first and key step in the development of an atheroma is endothelial dysfunction, which allows lipid infiltration and triggers an inflammatory response within the vascular wall. Following endothelial dysfunction, subendothelial retention of LDL is facilitated by arterial wall proteoglycans, whose negatively charged glycosaminoglycan chains bind to apoB-100, leading to prolonged lipid retention within the intima [2,3]. This retained LDL undergoes oxidative modification, thereby promoting local inflammation and initiating atherogenic processes.

Beyond epicardial atherosclerosis, CAD may also involve coronary microvascular dysfunction, where myocardial perfusion is impaired without significant epicardial stenosis. Coronary microvascular dysfunction is particularly relevant in patients with primary cardiomyopathies, myocarditis, and heart failure (HF), particularly heart failure with preserved ejection fraction (HFpEF) [4,5]. Coronary artery calcification (CAC) is a hallmark of advanced atherosclerosis and serves as a direct indicator of coronary artery disease burden and distribution. Quantitative assessment of calcification, typically via noninvasive imaging, provides robust evidence of underlying disease and is a powerful predictor of future cardiovascular events, outperforming traditional risk factors and other noninvasive biomarkers in risk stratification [6]. The presence and extent of calcifications are now integrated into clinical risk assessment algorithms and inform allocation of preventive therapies [7]. However, the mechanistic link between calcification and plaque vulnerability remains complex. While higher overall calcification burden correlates with increased risk of events due to greater total plaque volume, specific calcification patterns, such as spotty or microcalcifications, are more frequently associated with unstable plaques, whereas dense, sheet-like calcification is characteristic of stable lesions [8]. This paper explores the mechanisms driving atherosclerosis, with a particular focus on the initiation and progression of vascular calcification. It highlights key aspects of pathogenesis, pathophysiology, diagnostics, prevention, treatment and clinical implications, offering an integrated perspective on how plaque development and calcification contribute to the burden of cardiovascular disease.

## 2. Epidemiology

Coronary artery calcification is a well-established marker of subclinical atherosclerosis and is commonly detected in the general adult population. Its prevalence and burden increase almost linearly with age and are consistently higher in men than in women across all age groups. In large population-based cohort studies, about 16% of asymptomatic adults older than 30 years develop detectable CAC within 5–6 years of follow-up. In contrast, clinically significant calcification (CAC score > 100) is relatively uncommon, occurring in only around 4% of individuals even after 10 years of follow-up. In middle-aged and older adults, both the prevalence and severity of CAC increase with age, and CAC is commonly detected in individuals older than 80 years [9,10].

## 3. Classical Cardiometabolic Determinants

Chronological aging remains the predominant non-modifiable determinant of CAC burden, demonstrating a near-linear association with both prevalence and progression across all demographic strata. Sex differences are evident, with males exhibiting higher calcific burden at any given age; however, estrogen deficiency following menopause markedly accelerates CAC progression in women. Arterial hypertension, characterized by sustained elevations in systolic and diastolic blood pressure, promotes endothelial injury, vascular smooth muscle cell (VSMC) transdifferentiation, and subsequent calcium deposition [11].

Recent Mendelian randomization (MR) studies and MR-based meta-analyses have provided strong genetic evidence supporting a causal role of LDL-C and apoB-containing lipoproteins in CAD and atherosclerotic burden, reinforcing the mechanistic link between dyslipidemia and CAC. Genetically determined elevations in LDL-C are consistently associated with increased CAD risk, while multivariable MR analyses identify apoB as the principal lipid-related determinant of atherosclerotic cardiovascular disease, outperforming other lipid and triglyceride-related metrics [12].

**Figure 1:**
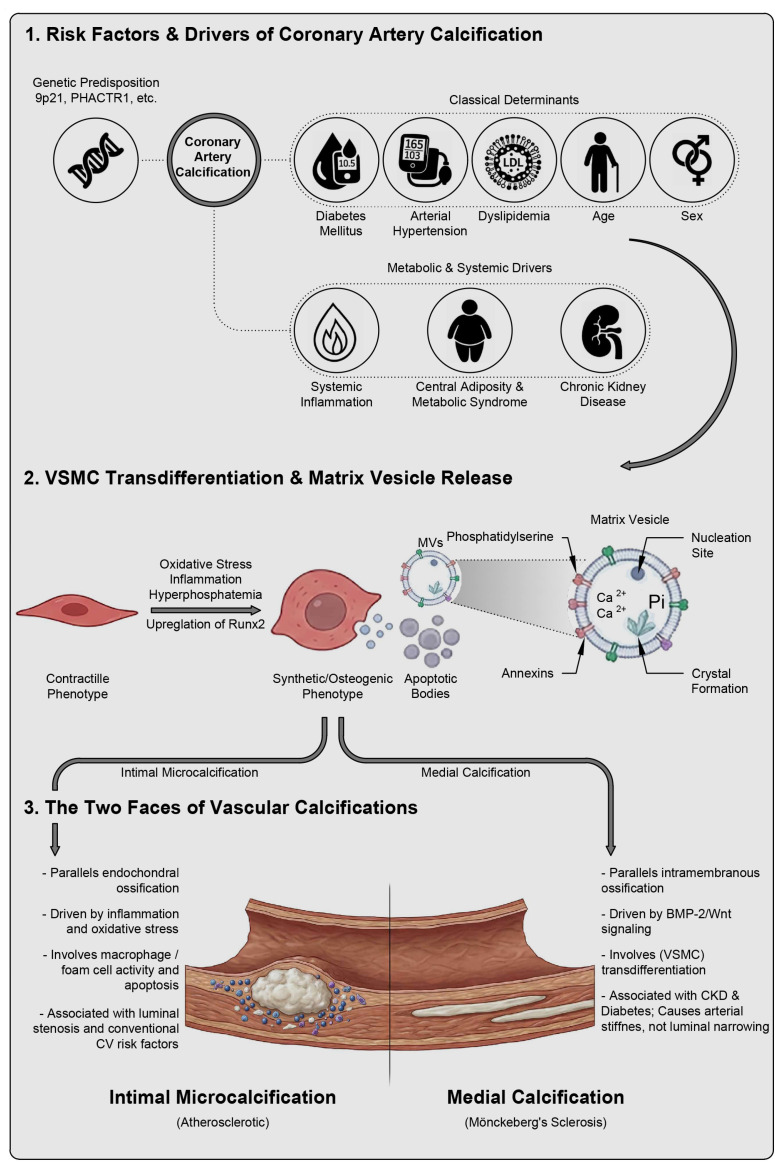
Integrated pathophysiology of vascular calcification: risk factors, cellular mechanisms, and pathohistological manifestations. VSMC—vascular smooth muscle cell; MVs—matrix vesicles; CV—cardiovascular; CKD—chronic kidney disease.

Furthermore, genetic causal inference studies support significant associations between type 2 diabetes mellitus, insulin resistance, obesity, hypertension, and both coronary atherosclerosis and CAC progression, emphasizing the multifactorial cardiometabolic basis of vascular calcification [13] (Figure 1).

Central adiposity and the metabolic syndrome (characterized by insulin resistance, dyslipidemia, hypertension, and low HDL-C) confer heightened CAC risk through adipokine imbalance, low-grade inflammation, and endothelial dysfunction. A familial predisposition to premature atherosclerotic cardiovascular disease (ASCVD) further augments risk through shared genetic and epigenetic susceptibilities affecting lipid metabolism and vascular calcification pathways [14]. Chronic systemic inflammatory disorders, including rheumatoid arthritis, systemic lupus erythematosus, psoriasis, and HIV infection, amplify CAC formation via persistent immune activation, cytokine overexpression, and endothelial dysfunction. Nontraditional risk enhancers, such as premature menopause, gestational metabolic disturbances (preeclampsia, gestational diabetes), peripheral arterial disease, South Asian ancestry, and elevated high-sensitivity C-reactive protein (hs-CRP), further modulate susceptibility to vascular calcification [14]. In chronic kidney disease (CKD), vascular calcification represents a hallmark of uremic vasculopathy. Perturbations in calcium-phosphate homeostasis, mediated by elevated fibroblast growth factor-23 (FGF-23), parathyroid hormone (PTH), and serum phosphate, drive osteogenic transformation of VSMCs. Concurrently, reduced glomerular filtration and chronic systemic inflammation (e.g., interleukin-6, TNF-α activation) accelerate calcific deposition within the coronary arterial wall [13,15,16] (Figure 1).

## 4. Genetic Determinants

Coronary artery calcification is a highly heritable phenotype and a robust marker of subclinical atherosclerosis, with genetic factors playing a significant role in its development and progression. Large-scale genome-wide association studies (GWAS) have identified multiple loci associated with CAC, including both loci shared with CAD and loci unique to CAC. Notably, the 9p21 region (CDKN2B-AS1) is consistently associated with CAC across diverse populations, including European and Korean cohorts, and is also implicated in CAD risk [17]. Recent multi-ancestry GWAS meta-analyses have expanded the number of known CAC risk loci, identifying genes involved in bone mineralization, phosphate metabolism, and hormone pathways, which are distinct from those driving clinical CAD. These findings highlight the biological overlap and divergence between CAC and CAD genetics. Functional studies support the role of these loci in smooth muscle cell-mediated calcification, further elucidating the pathophysiology of vascular calcification [17]. Other loci such as PHACTR1, COL4A2, and ATP2B1 have demonstrated associations with both CAC and myocardial infarction, suggesting that genetic influences on CAC are mechanistically linked to atherosclerotic disease progression [18] (Figure 1).

## 5. Mechanisms of Calcification

Arterial calcification arises from the crystallization of calcium and phosphate as hydroxyapatite within the arterial extracellular matrix. Based on the primary localization, it is classified into two main forms: medial calcification (known as Mönckeberg’s sclerosis) and intimal atherosclerotic calcification. Although each of these types of calcifications have distinct etiologies and pathophysiological implications, cells having a key role in both mechanisms are vascular smooth muscle cells (VSMC) [19]. These cells exhibit phenotypic plasticity, meaning that they can change their gene expression, function and appearance according to the environmental factors. Predominantly, VSMCs have a contractile phenotype, expressing high levels of contractile proteins, such as α-smooth muscle actin (α-SMA), smooth muscle myosin heavy chains (SM-MHC), calponin, smoothelin, etc. However, under pathological circumstances, VSMCs may undergo a phenotypic shift, lose their contractile properties and obtain secretory, osteoblast-like, or chondrocyte-like phenotype. The SMAD pathway, activated downstream of BMP signaling (particularly BMP-2), plays a central role in arterial calcification by promoting the osteogenic reprogramming of VSMC. Through phosphorylation of SMAD1/5/8 and nuclear translocation with SMAD4, it induces expression of osteogenic genes such as Runx2, alkaline phosphatase, and osteocalcin while suppressing the contractile phenotype, thereby driving a bone-like mineralization process within the vascular wall [20].

Nuclear factor κB (NF-κB) is a key inflammatory transcription factor that links oxidative stress and cytokine signaling to VSMC phenotypic switching and osteogenic reprogramming, thereby promoting vascular calcification through induction of osteogenic mediators such as Runx2 and alkaline phosphatase and through interaction with BMP–SMAD and Wnt signaling pathways that amplify mineralization programs in both intimal and medial vascular lesions [21].

### 5.1. Medial Arterial Calcification

Medial arterial calcification (MAC) is characterized by transdifferentiation of VCSM into osteoblast-like cells, which is mediated by BMP-2/MSX/Wnt signaling and represents a characteristic of intramembranous ossification [22]. In contrast to intimal calcification, MAC occurs independently of atherosclerotic plaque formation and is predominantly associated with systemic metabolic dysregulation, including diabetes mellitus, chronic kidney disease, and advanced age. It is known that the major drivers of osteogenic transdifferentiation of VSMCs are hyperphosphatemia, hyperglycemia, and pro-inflammatory as well as pro-coagulant signaling pathways [23,24]. Under such circumstances, there is an upregulation of bone matrix proteins (osteocalcin, osteopontin, and alkaline phosphatase) as well as an increase in ECM components, which are suitable for calcium deposition. These osteoblast-like cells release matrix vesicles that will serve as nucleation sites. Progressive medial arterial calcification results in hemodynamic changes due to increased arterial stiffness and loss of viscoelastic compliance. These biomechanical alterations elevate the arterial compliance and pulse wave velocity, augment systolic pressure, and impair diastolic recoil, resulting in diminished peripheral perfusion, tissue hypoxia, and increased left ventricular afterload. Unlike intimal (atherosclerotic) calcification, MAC does not result in luminal narrowing; rather, it disrupts arterial elasticity and wave reflection dynamics, thereby exacerbating hemodynamic load and complicating endovascular interventions through reduced procedural efficacy and long-term patency [24,25]. Histologically, MAC is characterized by diffuse, linear and often circumferential calcification within the medial layer, often associated with elastin fiber degradation and thinning of the tunica media [26,27].

### 5.2. Intimal Calcification

Intimal calcification is closely connected to atherosclerosis, where inflammatory and oxidative stress signaling drives transdifferentiation of VSMC into a chondrocyte-like phenotype [28]. This process is controlled by pro-osteochrondrogenic pathways (such as BMP, TGF-β, ERK/MAPK, etc.), where VSMC express SOX9, which promotes chondrocyte differentiation and chondrogenesis [29]. Due to its close association with atherosclerosis, conventional cardiovascular risk factors, including hyperlipidemia, hypertension, and smoking, predominate in the pathogenesis of intimal calcification [30]. Apoptosis of inflammatory cells within the atheromatous core and the subsequent liberation of apoptotic bodies may serve as nucleation sites for calcium crystal formation [31]. Other important sources of nucleation sites in intimal calcification are matrix vesicles (MVs)—small, membrane-bound extracellular vesicles released by VSMCs and macrophages. They play a central role in the initiation and propagation of vascular calcification, including coronary arteries [32]. They act as nucleation sites for hydroxyapatite crystal formation by concentrating calcium and phosphate within their lumen and on their membrane surfaces, thereby facilitating the earliest steps of mineral deposition in the extracellular matrix [33]. In the context of CAC, MVs are released in response to cellular stressors such as apoptosis, oxidative stress, inflammation, and disturbed calcium homeostasis, processes commonly seen in atherosclerosis, diabetes, and chronic kidney disease [32,34]. VSMC-derived MVs undergo phenotypic changes under these conditions, losing key mineralization inhibitors (e.g., matrix Gla protein) and becoming enriched in pro-calcific proteins such as annexins (especially AnxA6) and phosphatidylserine, which together provide a scaffold for hydroxyapatite nucleation. The exposure of phosphatidylserine on the MV surface is a critical event, as it binds calcium and promotes crystal formation [35]. Osteocalcin-positive endothelial progenitor cells, exhibiting an osteogenic phenotype, contribute to the development and progression of CAC by promoting aberrant vascular repair and early ossification; their elevated levels in patients with advanced atherosclerosis suggest a potential mechanistic and biomarker role in vascular and valvular calcification [36] (Figure 1).

Histologically, intimal calcification is often patchy and eccentric, and often associated with lipids, inflammatory cells, and neointimal thickening. This type of calcification may vary in size and therefore be categorized into: microcalcifications (0.5–15 µm), punctate (15 µm–1 mm), fragmentary (>1 mm), sheet (>3 mm), and nodular types. Nodular calcifications are thought to arise from mechanical disruption of pre-existing calcified sheets. When non-eruptive, they may extend into the medial layer without compromising the fibrous cap, primarily contributing to luminal narrowing [37,38]. In contrast, eruptive calcified nodules are a distinct form of advanced atherosclerotic disease in which dense calcium protrudes into the coronary lumen and disrupts the fibrous cap. Their eruption can provoke intraluminal thrombosis, making them an important but less common mechanism of acute coronary thrombosis and sudden cardiac death [39]. Calcification emerges early in atherosclerotic disease but becomes radiographically evident only after significant vascular deposition. A strong correlation exists between coronary calcium load and atherosclerotic burden, though not all plaques are calcified [40].

Intimal calcification often correlates with luminal stenosis and is associated with larger plaque burden, intraplaque hemorrhage, and increased risk of ischemic events [41].

### 5.3. Role of Pericytes in Arterial Calcification

Arterial calcification is now regarded as an active, tightly regulated process resembling bone formation rather than a passive calcium deposition. In this context, pericytes are increasingly recognized as important contributors to their pathogenesis, as multipotent mesenchymal cells associated with the microvasculature that may also be found within vascular wall compartments under pathological conditions. In response to pro-calcific stimuli, pericytes and related progenitor populations can undergo osteogenic differentiation, forming mineralized nodules and expressing key bone markers such as osteopontin, osteocalcin, osteonectin, and bone sialoprotein [42]. In human atherosclerotic plaques, cells within calcified regions display pericyte-like features and express BMP-2, a central osteogenic mediator, supporting their role in regulated vascular “osteogenesis” [43]. Pericytes present in the intima, media, and adventitia may thus act as a mesenchymal reservoir contributing to the osteogenic switch within the bone–vascular axis [44].

Beyond direct differentiation, pericytes actively shape the vascular microenvironment. Their increased presence in calcified, osteoid-rich plaques and their ability to modulate osteogenic signaling in neighboring cells underscore a broader regulatory role. They may also contribute to lipid uptake, foam cell formation, and local inflammation, which are key drivers of calcification [44]. Conversely, pericyte dysfunction or loss is associated with intraplaque angiogenesis, hemorrhage, and progression of calcification, features of plaque instability. Overall, pericytes integrate inflammatory, metabolic, and osteogenic signals, positioning them as important regulators of arterial calcification and potential therapeutic targets in atherosclerotic disease.

## 6. Diagnostics of Coronary Artery Calcifications

### 6.1. Laboratory Findings

Recent studies have explored the potential of specific serum biomarkers as predictors of CAC, including its distribution pattern and rate of progression. Alkaline phosphatase (ALP) is an enzyme that regulates phosphate metabolism and hydrolyzes pyrophosphate, a key inhibitor of vascular calcification. Elevated serum ALP levels have been associated with increased CAC and greater atherosclerotic burden, independent of traditional cardiovascular risk factors. An association is also evident for individuals with normal phosphate levels and in the absence of chronic kidney disease, challenging the concept that only marked hyperphosphatemia in the setting of chronic kidney disease promotes calcification. This relationship suggests that ALP may not only serve as a biomarker of vascular injury and inflammation but may also play a direct role in promoting calcification within the arterial wall [45]. Recent evidence further supports this concept, showing that higher serum ALP levels are independently associated with greater coronary calcium burden, spotty calcification, and vulnerable plaque features, indicating a potential mechanistic role of ALP in plaque instability and cardiovascular risk [46]. Elevated lipoprotein (a) (Lp(a)) is a genetically determined, proatherogenic, proinflammatory, and prothrombotic lipoprotein particle that promotes atherogenesis and vascular calcification through mechanisms such as stimulation of osteogenic differentiation in vascular smooth muscle cells and induction of vascular inflammation [47]. Recent large cohort studies and meta-analyses demonstrate that high Lp(a) levels are associated with a greater likelihood of having any CAC (CAC score > 0), more advanced calcification (CAC score ≥ 100), and faster progression of CAC over time, although the strength of this association is modest and subject to heterogeneity [48]. Emerging research has investigated novel circulating biomarkers, such as microRNAs (e.g., miR-126-3p and miR-145-5p), which have shown some diagnostic value for arterial calcification in small studies. For example, lower plasma levels of miR-126-3p and miR-145-5p were associated with the presence of coronary calcification and adverse cardiovascular events in a recent cohort, suggesting potential utility as blood-based biomarkers [49]. However, these findings are preliminary, and such biomarkers are not currently validated or recommended for routine clinical use in the detection of CAC. Other serum biomarkers associated with CAC include irisin (inversely related to CAC progression), matrix metalloproteinases-2 and -9 (promoting matrix degradation and calcification), 1,5-anhydro-D-glucitol (inversely linked to calcium burden in diabetes), uric acid (associated with vulnerable plaque features), and osteogenic monocytes (implicated in early atherogenesis and calcified plaque formation), though their potential roles in the pathogenesis and progression of CAC remain to be fully established [31]. Beyond these, a range of novel biomarkers, including Tenascin-C, IL-37, PTX3, transthyretin, soluble interleukin-6 receptor α, and miR-15a, have shown high diagnostic potential for chronic coronary artery disease in recent reviews, but their specific association with CAC requires further validation in prospective studies [50]. However, while these biomarkers provide valuable insights into the active biological mechanisms of atherogenesis, they do not replace the superior prognostic accuracy of direct CAC quantification, which remains the clinical benchmark for long-term cardiovascular risk stratification, given that its predictive power has been robustly validated across different clinical registries [51,52].

### 6.2. Computed Tomography

Computed tomography (CT) is the standard non-invasive method for detecting and characterizing calcified coronary lesions. Coronary CT angiography (CCTA) provides information on both luminal stenosis and plaque morphology, including the presence, extent, distribution, and density of calcifications [53]. Tissue differentiation in CT is based on X-ray absorption and attenuation, which determines grayscale appearance on reconstructed images. Attenuation is expressed in Hounsfield units (HU), introduced by Hounsfield following the development of the first CT scanner [54,55]. Calcium exhibits high attenuation due to its density and atomic number, appearing bright and sharply defined on CT images. Modern CT scanners, including dual-source and high-definition systems, improve temporal and spatial resolution, allowing better visualization of small calcific deposits and partial volume effects.

**Figure 2:**
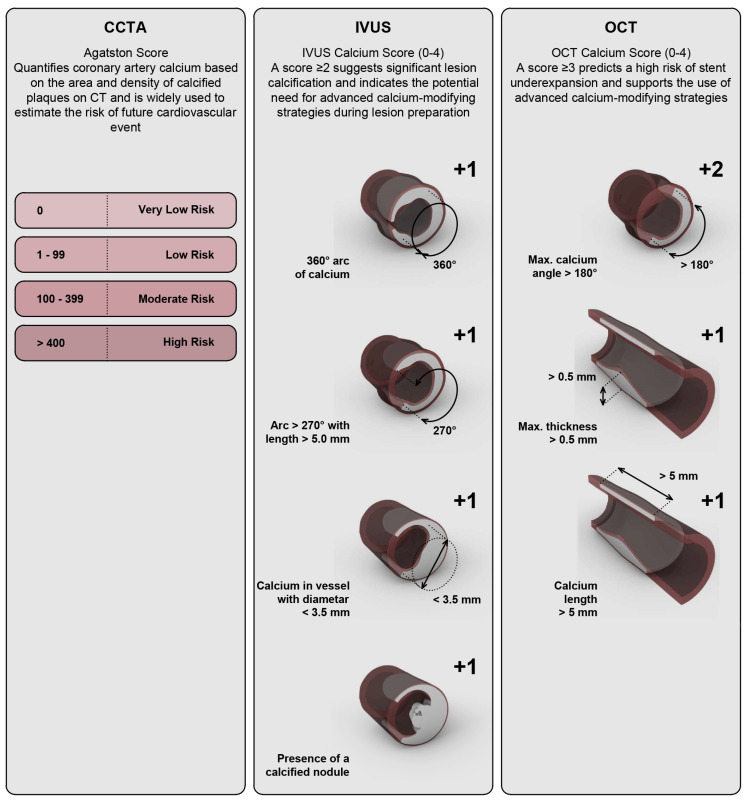
Coronary artery calcium scores derived from different imaging modalities. CCTA—coronary CT angiography; IVUS—intravascular ultrasound; OCT—optical coherence tomography.

Coronary calcification is a marker of atherosclerosis and carries prognostic value [56]. The Agatston score (Figure 2) remains the most widely used method for quantifying calcium burden, incorporating both plaque density and area. Scores of 0 indicate absence of detectable calcium, while values > 400 reflect extensive calcification and high cardiovascular risk [57]. Beyond quantification, CCTA identifies plaque features associated with vulnerability in non-flow-limiting plaques, also known as thin-cap fibroatheromas (TCFA) [58]. These include spotty calcifications, low-attenuation plaques, positive remodeling, and the “napkin-ring” sign [59]. Such features are linked to an increased likelihood of future acute coronary syndromes, and emerging evidence suggests that preventive percutaneous intervention in selected cases may reduce adverse outcomes [60].

Coronary CT angiography improves understanding of coronary anatomy, lesion complexity, and plaque distribution, facilitating procedural planning, especially in challenging scenarios such as chronic total occlusions [61]. Calcium density assessment on CT may also help guide the use of dedicated calcium-modifying strategies, including rotational atherectomy, orbital atherectomy, intravascular lithotripsy, or cutting/scoring balloons [62]. However, dense calcification may limit CT accuracy due to blooming and beam-hardening artifacts, which can overestimate the extent of lesions and hinder precise assessment of luminal stenosis. In patients with high calcium burden, complementary modalities such as invasive coronary angiography (ICA), intravascular imaging, or physiological assessment are often necessary to define true anatomical or functional significance.

Imaging-derived biomarkers are also evolving: the perivascular fat attenuation index (pFAI), measured by CCTA, is emerging as an indirect marker of coronary inflammation and may complement CAC scoring for risk stratification [63]. Additionally, aortic calcification detected on chest radiography or CT is strongly associated with CAC and may serve as a surrogate marker for ischemic heart disease risk [64].

### 6.3. Other Non-Invasive Imaging Techniques

Other non-invasive imaging techniques, such as Positron Emission Tomography (PET) and Magnetic Resonance Imaging (MRI), can also be used as complementary tools in assessing coronary calcification, mainly by evaluating the pathophysiological context of calcified plaques. Because of its ability to detect active calcification at the lowest molecular level, the ^18^F-NaF (^18^F-sodium fluoride) PET has emerged as a powerful method to predict the progression of coronary calcification, thereby identifying patients at a significantly higher risk for future adverse cardiovascular events [65]. MRI has also proven to be accurate in identifying coronary calcification under ex vivo conditions [66]; however, its translation into routine clinical practice remains limited. This is primarily due to technical constraints and the inherent signal voids caused by calcium; consequently, the clinical focus of in vivo MRI has shifted from direct calcium quantification toward evaluating vessel wall thickness and overall plaque vulnerability [67].

### 6.4. Invasive Coronary Angiography

Invasive coronary angiography (ICA) remains the most widely used invasive method for detecting coronary atherosclerosis. Calcium can be visualized angiographically, but typically only when extensive and dense (length > 6 mm, eccentricity > 180° [68]. Angiography provides a luminogram, and as such, underestimates the presence and severity of calcium. The degree of coronary calcification is an important determinant of lesion complexity and is incorporated into evaluation. To meet this criterion, calcified densities must be visible in more than one projection and circumferentially surround the coronary lumen at the lesion site [69]. Extensive calcification increases procedural difficulty, may limit optimal stent expansion, and is associated with a higher risk of periprocedural complications [70]. Mild or moderate calcification, however, is frequently not detected, necessitating adjunctive imaging such as intravascular ultrasound (IVUS) or optical coherence tomography (OCT) for accurate characterization [68,71].

### 6.5. Intravascular Ultrasound

Intravascular ultrasound is a well-established intracoronary imaging technique used to detect and characterize CAC with far greater sensitivity than ICA. Intravascular ultrasound provides cross-sectional visualization of the arterial wall and plaque composition. On IVUS, coronary calcium appears as a highly echogenic region with posterior acoustic shadowing, enabling detection of both superficial and deep calcific deposits often missed by angiography. In addition to identifying calcium, IVUS quantifies its arc, depth, and longitudinal extent, parameters that are clinically relevant, as the severity and distribution of calcification significantly affect percutaneous coronary intervention (PCI) complexity and outcomes [72,73].

Calcium severity assessed by IVUS can be quantified using dedicated scoring systems, such as the score proposed by Zhang et al. (Figure 2), which assists in selecting lesions that may benefit from advanced calcium-modifying strategies. This score incorporates several lesion characteristics, including a 360° arc of calcium, a calcium arc greater than 270° with a calcium length of at least 5.0 mm, the presence of calcium in vessels with a diameter < 3.5 mm, and the presence of a calcified nodule [74].

Therefore, IVUS plays a critical role in procedural planning by helping determine the need for advanced calcium-modifying strategies. IVUS is equally valuable after lesion preparation and stenting. It enables confirmation of calcium modification (e.g., evidence of plaque fracture), as well as evaluation of stent expansion and apposition, which are essential for optimizing procedural success and improving long-term clinical outcomes [75,76,77]. Compared with angiography guidance alone, IVUS-guided PCI in calcified lesions is linked to superior procedural success rates [78]. Using IVUS in complex PCI, especially left main disease, has a class IA recommendation in the current European Society of Cardiology guidelines for the management of chronic coronary syndrome [79].

### 6.6. Optical Coherence Tomography

Optical coherence tomography is a high-resolution intracoronary imaging technique that provides detailed visualization of coronary calcification with superior axial resolution compared to IVUS. Calcified plaques on OCT are identified as low-signal, signal-poor regions with sharply delineated borders, allowing precise differentiation from fibrous or lipid-rich tissue components. The high resolution of OCT enables accurate measurement of calcium thickness, depth, arc, and longitudinal extent, offering detailed insight into plaque morphology [80,81,82]. These lesion characteristics contribute to lower procedural success when performing PCI, mainly due to stent under-expansion. OCT-derived scores exist to predict the probability of stent under-expansion. The commonly used OCT score (Figure 2) consists of three components: maximum thickness > 0.5 mm (1 point); contiguous length of calcium > 5 mm (1 point); and maximum calcium arc > 180° (2 points). A score of ≤3 is linked with efficient stent expansion, while higher score lesions usually demand some calcium-modification methods to achieve adequate stent expansion [83].

Using OCT as well as IVUS is associated with a better clinical outcome in comparison with angiography-guided PCI [84,85] and is encouraged in the current clinical guidelines [79]. OCT can help anticipate how calcified plaques will respond to calcium-modifying techniques by identifying patterns of calcium that are more likely to fracture during treatment [86]. Owing to its superior spatial resolution, it is also the most reliable method for detecting changes in calcium burden, lumen enlargement, and the presence of calcium fractures after modification [87].

## 7. Treatment and Prevention of Coronary Artery Calcifications

Calcified coronary lesions represent one of the most challenging subsets in interventional cardiology. The presence of calcium within the vessel wall limits balloon expansion, impedes optimal stent delivery and expansion, and increases the risk of complications such as dissection, perforation and stent underexpansion, which can result in restenosis or thrombosis [75]. As previously mentioned, there are several methods to diagnose the presence and the extent of CAC. Two mostly used methods in interventional cardiology are IVUS and OCT. Both modalities can estimate the arc and length of coronary calcium, but only OCT can accurately measure the thickness of calcium [74,83].

**Figure 3:**
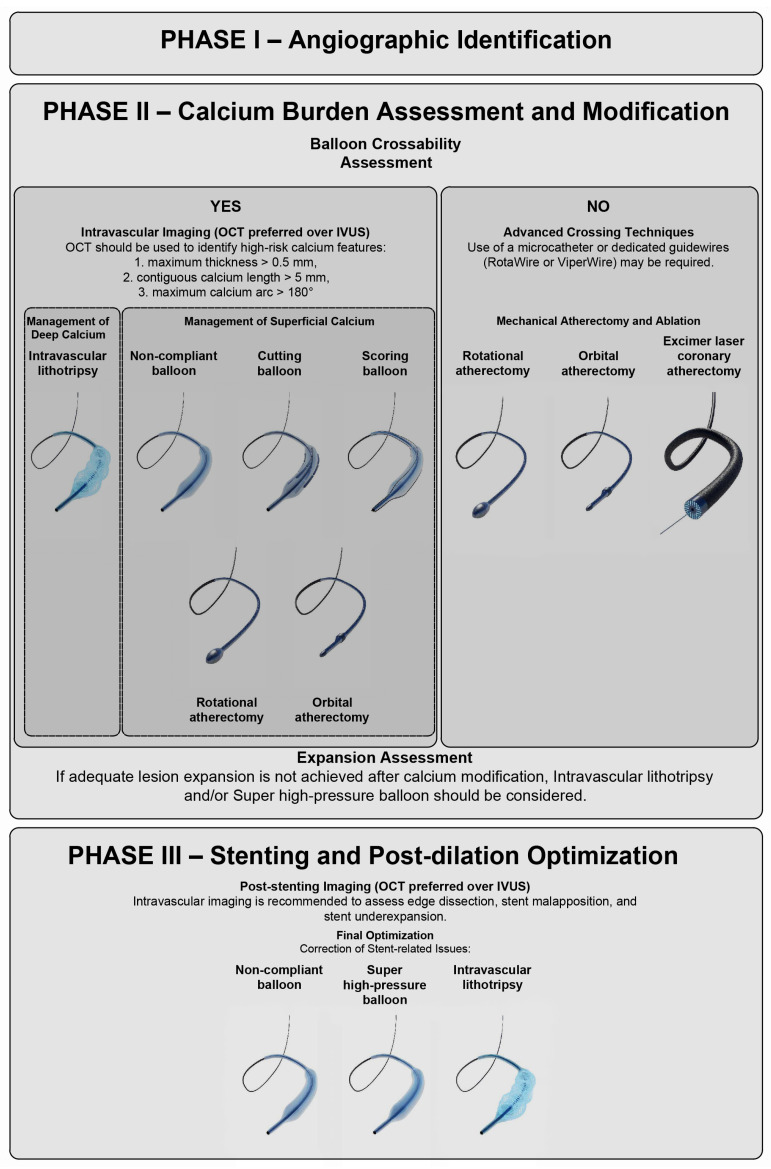
Algorithm for the management of calcified coronary lesions. OCT—optical coherence tomography; IVUS—intravascular ultrasound.

The goal of calcium modification is to achieve sufficient plaque compliance to allow optimal stent expansion while minimizing procedural risks [75,76]. A variety of devices can be used for this purpose, ranging from conventional balloon-based techniques (semicompliant (SC), noncompliant (NC), cutting, scoring, and super high-pressure balloons) to advanced calcium-modification technologies (rotational atherectomy (RA), orbital atherectomy (OA), excimer laser coronary atherectomy (ELCA), and intravascular lithotripsy (IVL)). Several clinical algorithms help determine which modality is most appropriate for a given lesion (Figure 3).

Balloon angioplasty with SC and NC balloons is mostly used for mildly calcified coronary lesions or to prepare severely calcified lesions for further treatment. There are several limitations associated with the use of these balloons. Their expansion at the site of a calcified lesion may be eccentric (toward the area of least resistance), leading to the so-called dog-bone shape, which can cause dissection or perforation. Additionally, balloon slippage across the lesion may occur, and finally, rupture of the balloon caused by calcium spikes can result in vessel perforation [88].

There are several types of scoring balloons used as tools for calcium modification. Their common feature is the presence of one or more protruding metallic elements on the balloon surface that encounter the vessel wall during inflation, scoring the plaque and facilitating better lumen expansion compared to standard balloon angioplasty [89].

Cutting balloons are NC balloons equipped with three to four microsurgical blades along their longitudinal surface. During low-pressure inflation, these blades create precise, shallow incisions within the calcified plaque, facilitating controlled calcium fracture and thereby enabling more uniform balloon and stent expansion [90].

Super high-pressure balloons are dual-layered, NC balloons that can be inflated up to 40 atmospheres (atm) with greater uniformity than other balloons. One of the most used is the OPN balloon (SIS Medical, Switzerland). Secco et al. have conducted a retrospective analysis, which showed that when NC balloons fail, OPN provides an effective strategy to dilate resistant calcified lesions. It was also observed that acute luminal gain was significantly higher with OPN, with greater procedural success and no coronary perforations observed [91]. It is also shown that super high-pressure balloons can be effectively used for stent postdilation. Although these balloons are highly effective for lesion preparation and postdilation, it is important to recognize that the endothelial injury they potentially cause serves as a substrate for subsequent restenosis [92,93].

Intravascular lithotripsy is performed using the Shockwave Intravascular Lithotripsy System, which consists of a balloon catheter with integrated emitter pairs that generate pulsatile sonic shockwaves. These waves interact with calcium deposits (both superficial and deep) within the vessel wall, creating micro and macrofactures that facilitate stent expansion. When the sonic waves interact with calcium, they amplify to an effective pressure of approximately 50 atm. The Shockwave balloon is typically inflated to 4 atm at the lesion site and delivers 10 shockwaves per cycle, with a maximum of 120 shockwaves per lesion [94]. The Disrupt CAD III study, a prospective, single-arm, multicenter trial including 384 patients, demonstrated a high procedural success rate with a low rate of complications. Although not designed for comparative evaluation, the trial confirmed the safety and effectiveness of IVL for lesion preparation in severely calcified coronary arteries. However, we acknowledge that its single-arm, non-randomized design and relatively modest sample size may introduce statistical biases, and therefore these findings should be interpreted with caution [95].

Rotational atherectomy is a specialized lesion modification technique that utilizes a high-speed rotational catheter equipped with a diamond-tipped burr to ablate calcified coronary plaques. The micro-fragments generated during this process, typically measuring 2.0 to 10.0 µm in diameter, safely pass through the microcirculation and are subsequently cleared by the reticuloendothelial system [96]. The ROTAXUS trial, which randomized 240 patients with calcified coronary lesions to PCI either with or without upfront RA, demonstrated that routine atherectomy significantly improved acute lumen gain (1.56 ± 0.43 mm vs. 1.44 ± 0.49 mm, *p* = 0.01) and substantially increased the likelihood of successful stent implantation (92.5% vs. 83.3%, *p* = 0.03; OR 2.52, 95% CI 1.07–5.94) [97]. Similarly, the PREPARE-CALC trial randomized 200 patients with severely calcified lesions to receive either RA or specialized cutting/scoring balloon therapy. Although RA achieved a markedly higher procedural success rate (98% vs. 81%, *p* = 0.0001; RR 1.21, 95% CI 1.10–1.33), this technical superiority did not translate into a significant reduction in clinical endpoints at the 9-month follow-up timeframe. Specifically, no statistically significant differences were observed regarding target vessel failure (TVF: 8% versus 6%; *p* = 0.78) or target lesion revascularization (TLR: 7% vs. 2%; *p* = 0.17). Furthermore, the primary endpoint of in-stent late lumen loss (LLL) at 9 months remained comparable between the two cohorts (0.22 ± 0.40 mm vs. 0.16 ± 0.39 mm, *p* = 0.21) [98]. The orbital atherectomy system consists of an eccentrically mounted 1.25 mm diamond-coated crown, enabling bidirectional atherectomy, a distinct advantage over rotational RA, which is limited to forward ablation. The mechanism of action relies on centrifugal force and surface friction, which generate elliptical orbits and produce microfractures in the calcified plaque. Importantly, this reliance on orbital motion rather than simple forward mechanical force allows for the effective modification of very tight or uncrossable lesions. As the rotational speed increases, the elliptical trajectory expands the effective ablation area while simultaneously permitting continuous blood flow and efficient debris clearance during the procedure [99]. The ORBIT II trial was a prospective, multicenter, single-arm study evaluating OA in 443 patients with severely calcified coronary lesions. It showed a high procedural success rate and met its predefined safety and efficacy endpoints, supporting OA as an effective strategy for lesion preparation in this population [100].

Excimer laser coronary atherectomy delivers high-energy ultraviolet pulses that disrupt carbon bonds within plaque. Its effect is mediated through three complementary mechanisms: 1. ablation—direct molecular bond disruption, 2. cavitation—formation and collapse of fluid bubbles that debulk plaque, and 3. acoustic mechanical impact—shockwaves that create microfractures. Clinically, ECLA is mainly applied in chronic total occlusions, saphenous vein graft interventions, and calcified in-stent restenosis, rather than in heavily calcified de novo lesions [101]. Its limited utility in severe calcification was demonstrated in the ERBAC trial, which compared excimer laser, balloon angioplasty, and rotational atherectomy. Although rotational atherectomy achieved higher procedural success, there were no significant differences in major in-hospital complications between treatment groups [102].

A contemporary approach to managing calcified coronary lesions involves a sequential strategy that begins with initial angiographic screening, followed by a detailed assessment of plaque severity and characteristics using intravascular imaging. At this stage, OCT is generally preferred over IVUS as it allows for the precise identification of high-risk anatomical features [84].

Clinical decision-making then fundamentally depends on whether the lesion can be crossed with standard balloons and imaging catheters.

When the lesion is crossable, the therapeutic strategy is tailored to the depth of the calcium within the vessel wall. Superficial calcifications are effectively managed using NC balloons, specialized cutting or scoring balloons, or direct mechanical debulking with RA or OA [103]. Conversely, when calcified deposits are located within the deeper layers of the vessel wall and overlain by fibrotic tissue, they can still be modified with NC or specialty balloons; however, IVL is particularly effective in this scenario due to the transmural transmission of acoustic shockwaves that successfully fracture deep calcium [104].

In contrast, when the lesion is uncrossable for basic balloons or imaging catheters, advanced crossing techniques using microcatheters or dedicated guidewires, such as RotaWire or ViperWire, become necessary. In this setting, RA or OA represent the standard and widely available first-line strategies for mechanical ablation of the lesion entrance [105]. ELCA serves as a legitimate alternative in selected cases, performing front-cutting ablation over standard frontline wires to modify the lesion entry and create sufficient space for subsequent balloon passage [106]. If adequate lesion expansion is still not achieved after these steps, the use of IVL or super high-pressure balloons is recommended before stent implantation [103,104].

Finally, the definitive phase focuses on stenting and post-dilation optimization. Performing intravascular imaging after stenting is crucial to rule out edge dissections, malapposition, or underexpansion, while any stent-related issues are corrected through the stepwise deployment of NC balloons, super high-pressure balloons, or IVL [103,104,107].

Nowadays, a hybrid approach is increasingly being used, e.g., combining RA and IVL, the so-called “Rotatripsy”, as these modalities act on calcified plaques through different mechanisms, thereby achieving better results. The Rota-Shock registry, a prospective multicenter study of 160 patients, demonstrated that the sequential use of RA and IVL achieved a 96.9% procedural success rate with excellent safety outcomes. Freedom from serious angiographic complications occurred in 90.6% of cases, with low rates of perforation (2.5%), slow/no flow (5.0%), and major dissection (1.9%). Notably, in-hospital major adverse cardiac and cerebrovascular events (MACCE) occurred in only 1.3% of patients. This approach is particularly effective in complex cases where RA provides the necessary “debulking” to deliver an IVL balloon, which then addresses deeper calcific components through acoustic shockwaves [107,108,109].

Although interventional procedures represent the primary treatment for significant flow-limiting CAC, pharmacological approaches do not reverse calcifications but may influence cardiovascular risk in correlation with the extent of calcification [110]. Aspirin has been evaluated for its potential effect on CAC progression, but current evidence is inconclusive. Large cohort studies, including MESA, found no significant association between regular aspirin use and reduced CAC incidence or progression, although some data suggest a potential role for primary prevention in individuals with elevated CAC (≥100 Agatston units), high ASCVD risk, and low bleeding risk [111,112]. Statin therapy has been associated with increased plaque calcification due to a reduction in lipid content and promotion of plaque stabilization. Despite increases in CAC scores, statins reduce cardiovascular risk in patients with elevated CAC, particularly those with scores above 100, supporting their critical role in primary prevention [113]. PCSK9 inhibitors reduce LDL cholesterol and cardiovascular risk in patients who do not achieve target LDL levels with statins, but evidence regarding their effect on CAC progression is limited, and their use for CAC-specific risk modulation remains uncertain [114].

## 8. Clinical Implications

Calcified CAD is linked to poorer outcomes following revascularisation, with multiple PCI studies noting incomplete intervention in complex lesions, likely reflecting procedural caution. In asymptomatic individuals, a CAC score of zero, the “power of zero”, is the strongest negative predictor of cardiovascular events, surpassing traditional and emerging markers such as carotid intima-media thickness, absence of carotid plaque, family history, ankle-brachial index, B-type natriuretic peptide, albuminuria, and hs-CRP, and demonstrating superiority over polygenic risk scores for predicting 10-year coronary heart disease in large cohorts of primarily European ancestry [52,115].

The extent of CAC reflects total atherosclerotic burden and correlates with long-term risk of major adverse cardiovascular events (MACE), including myocardial infarction and mortality [116]. The relationship between calcification and plaque stability is nuanced: lesions causing acute coronary syndrome (ACS) or sudden cardiac death often feature necrotic, lipid-rich cores, positive remodeling, spotty calcifications, high plaque burden, severe stenosis, and thin-cap fibroatheromas [117]. Micro- or spotty calcifications within the fibrous cap amplify local stress and predispose plaques to rupture and thrombosis, though these are below the resolution of conventional CT and CAC scoring [38,118]. Therefore, high CAC indicates advanced atherosclerosis but does not reliably identify plaques likely to trigger ACS [38,117,118]. Fibroatheroma evolution, marked by progressive calcification and necrotic core reduction, is associated with plaque stabilization [119]. In chronic coronary syndromes, dense, confluent calcification characterizes stable fibrocalcific plaques, reducing rupture risk and reinforcing the fibrous cap, even as overall atherosclerotic burden increases [119,120].

CAC morphology substantially influences PCI complexity and outcomes. Concentric calcification (calcium arc >180°) increases procedural difficulty, impeding balloon dilation and stent expansion, and is linked to lower success rates, greater need for plaque modification, longer procedures, and higher perforation risk [121]. Lesions with calcium arcs > 270° or thickness > 500 μm generally require specialized devices such as rotational/orbital atherectomy, intravascular lithotripsy, or laser angioplasty [105,122]. Eccentric calcification (arc ≤ 180°) tends to be more amenable to conventional balloon angioplasty, though both morphologies can cause stent malapposition and risk of thrombosis or restenosis [70]. Recent data suggest intravascular lithotripsy efficacy is largely independent of calcium distribution, though concentric lesions may achieve more fractures per lesion [123]. Calcified nodules, especially eruptive types, are rarer but can complicate PCI due to higher rates of vessel dissection, even when stent expansion is adequate [123].

Moderate to severe CAC independently predicts adverse long-term outcomes post-PCI, with higher rates of MACE, including death, myocardial infarction, and repeat revascularization, at 3–5 years [124,125]. Target lesion failure risk rises with moderate/severe CAC [124,125,126], and in ACS, it increases stent thrombosis and TLR at 1 year [127]. In the SYNTAX study, 10-year mortality was higher for calcified versus non-calcified lesions [128]. Current artificial intelligence (AI)-based methods for detecting coronary calcifications, predominantly utilizing CTCA scans, are continuously evolving through the ongoing development and comparison of diverse algorithms, several of which have demonstrated promising results [129,130]. Furthermore, deep learning and neural networks have been applied to analyze invasive imaging modalities, such as OCT scans, demonstrating high accuracy in CAC detection [131]. Intracoronary imaging-guided calcium modification, using IVUS or OCT enhanced with AI for arc and depth quantification, offers precise lesion assessment and tailored intervention, improving stent expansion, reducing TLR, and enhancing long-term outcomes compared to angiography alone [84,132,133].

## 9. Conclusions and Future Perspectives

Coronary artery calcification, as a key marker of advanced atherosclerosis, reflects the overall burden of coronary artery disease and influences plaque stability as well as personal cardiovascular risk. The extent and morphology of calcification also significantly influence the technical challenges of PCI, frequently requiring specific strategies to ensure adequate myocardial revascularization. Advances in high-resolution imaging and patient-specific interventional planning show promise for improving procedural outcomes.

Looking forward, future perspectives lie in combining advanced molecular imaging (such as PET and MRI) and specific biomarkers to detect active, early-stage calcification before it becomes a structural challenge in the catheterization laboratory. Furthermore, a deeper understanding of how chronic pharmacological therapies affect vascular calcification pathways may open new doors for targeted early plaque modification. Ultimately, optimizing clinical management and long-term patient care requires a customized strategy that takes into consideration plaque phenotype and calcific burden.

## Data Availability

No new data were created or analyzed in this study. Data sharing is not applicable to this article.

## Abbreviations

The following abbreviations are used in this manuscript:

ACS	acute coronary syndrome
ALP	alkaline phosphatase
ASCVD	atherosclerotic cardiovascular disease
CAC	coronary artery calcification
CAD	coronary artery disease
CKD	chronic kidney disease
CT	computed tomography
CTCA	coronary CT angiography
ELCA	excimer laser coronary atherectomy
GWAS	genome-wide association studies
HDL-C	high-density lipoprotein cholesterol
HF	heart failure
HFpEF	heart failure with preserved ejection fraction
hs-CRP	high-sensitivity C-reactive protein
HU	Hounsfield units
HIV	human immunodeficiency virus
ICA	invasive coronary angiography
IVL	intravascular lithotripsy
IVUS	intravascular ultrasound
LDL-C	low-density lipoprotein cholesterol
Lp(a)	lipoprotein(a)
MAC	medial arterial calcification
MACE	major adverse cardiovascular events
MV	matrix vesicles
NC	noncompliant
OA	orbital atherectomy
OCT	optical coherence tomography
PCI	percutaneous coronary intervention
PCSK9	proprotein convertase subtilisin/kexin type 9
PTH	parathyroid hormone
RA	rotational atherectomy
SC	semicompliant
SMC	smooth muscle cell
TCFA	thin-cap fibroatheroma
TLR	target lesion revascularization
TNF-α	tumor necrosis factor alpha
VSMC	vascular smooth muscle cell
